# Supplementary material for: A human ESC-based screen identifies a role for the translated lncRNA LINC00261 in pancreatic endocrine differentiation
Source: eLife. 2020 Aug 3;9:e58659. doi: 10.7554/eLife.58659 (PMC7423336; doi:10.7554/eLife.58659)
Supplement: Transparent reporting form [file elife-58659-transrepform.docx]

***eLife’s* transparent reporting form**

We encourage authors to provide detailed information *within their submission* to facilitate the interpretation and replication of experiments. Authors can upload supporting documentation to indicate the use of appropriate reporting guidelines for health-related research (see [EQUATOR Network](http://www.equator-network.org/%20)), life science research (see the [BioSharing Information Resource](https://biosharing.org/)), or the [ARRIVE guidelines](http://www.plosbiology.org/article/info:doi/10.1371/journal.pbio.1000412) for reporting work involving animal research. Where applicable, authors should refer to any relevant reporting standards documents in this form.

If you have any questions, please consult our Journal Policies and/or contact us: [editorial@elifesciences.org](mailto:editorial@elifesciences.org).

**Sample-size estimation**

- You should state whether an appropriate sample size was computed when the study was being designed
- You should state the statistical method of sample size computation and any required assumptions
- If no explicit power analysis was used, you should describe how you decided what sample (replicate) size (number) to use

Please outline where this information can be found within the submission (e.g., sections or figure legends), or explain why this information doesn’t apply to your submission:

The numbers of replicates were chosen from experience with the variability inherent to the pancreatic *in vitro* differentiation system used in this study.

All relevant information can be found in the main text, methods and figure legends.

**Replicates**

- You should report how often each experiment was performed
- You should include a definition of biological versus technical replication
- The data obtained should be provided and sufficient information should be provided to indicate the number of independent biological and/or technical replicates
- If you encountered any outliers, you should describe how these were handled
- Criteria for exclusion/inclusion of data should be clearly stated
- High-throughput sequence data should be uploaded before submission, with a private link for reviewers provided (these are available from both GEO and ArrayExpress)

Please outline where this information can be found within the submission (e.g., sections or figure legends), or explain why this information doesn’t apply to your submission:

Numbers of replicate experiments are indicated in the relevant results, methods and figure legend sections. Outliers were not removed. Whether technical (e.g. pancreatic differentiation of the same CRISPR KO hESC lines) or biological replicates (e.g. pancreatic differentiation of CRISPR KO hESC generated with different pairs of sgRNAs) were used is defined in the relevant sections of the text. All mRNA-seq and Ribo-seq datasets generated for this study have been deposited at GEO under the accession number GSE144682. Data can be accessed at: https://www.ncbi.nlm.nih.gov/geo/query/acc.cgi?acc=GSE144682

**Statistical reporting**

- Statistical analysis methods should be described and justified
- Raw data should be presented in figures whenever informative to do so (typically when N per group is less than 10)
- For each experiment, you should identify the statistical tests used, exact values of N, definitions of center, methods of multiple test correction, and dispersion and precision measures (e.g., mean, median, SD, SEM, confidence intervals; and, for the major substantive results, a measure of effect size (e.g., Pearson's r, Cohen's d)
- Report exact p-values wherever possible alongside the summary statistics and 95% confidence intervals. These should be reported for all key questions and not only when the p-value is less than 0.05.

Please outline where this information can be found within the submission (e.g., sections or figure legends), or explain why this information doesn’t apply to your submission:

Statistical analyses were performed using Microsoft Excel, GraphPad Prism (7.05), and R (v.3.5.0). Statistical parameters such as the value of n, mean, standard deviation (S.D.), standard error of the mean (S.E.M.), significance level (*p < 0.05, **p < 0.01, ***p < 0.001 and ****p < 0.0001), and the statistical tests used are reported in the figures and figure legends. The ‘‘n’’ refers to the number of independent pancreatic differentiation experiments analyzed (biological replicates), or the number of genes/transcripts and sORFs detected.

Statistically significant gene expression changes were determined with DESeq2.

(For large datasets, or papers with a very large number of statistical tests, you may upload a single table file with tests, Ns, etc., with reference to sections in the manuscript.)

**Group allocation**

- Indicate how samples were allocated into experimental groups (in the case of clinical studies, please specify allocation to treatment method); if randomization was used, please also state if restricted randomization was applied
- Indicate if masking was used during group allocation, data collection and/or data analysis

Please outline where this information can be found within the submission (e.g., sections or figure legends), or explain why this information doesn’t apply to your submission:

Samples were allocated into either the control or knockout/frameshift group depending on the genotype of the hESC line used for pancreatic differentiation.

**Additional data files (“source data”)**

- We encourage you to upload relevant additional data files, such as numerical data that are represented as a graph in a figure, or as a summary table
- Where provided, these should be in the most useful format, and they can be uploaded as “Source data” files linked to a main figure or table
- Include model definition files including the full list of parameters used
- Include code used for data analysis (e.g., R, MatLab)
- Avoid stating that data files are “available upon request”

Please indicate the figures or tables for which source data files have been provided:

Source data files have been made available for all Figures. They provide a summary on all gene expression data (Figure 1-source data 1; Figure 2-source data 1), differential gene expression (Figure 3-source data 1; Figure 4-source data 1), gene expression correlation and gene ontology analysis (Figure 1-source data 1), translation (Figure 2-source data 1), Ribo-seq sample QC and ORFs (Figure 2-source data 1), qPCR results (Figure 3-source data 2-3), Insulin measurements (Figure 3-source data 4; Figure 4-source data 3), all primers, probes, synthetic genes and sgRNAs (Figure 4-source data 2).
